# Histopathology in focus: a review on explainable multi-modal approaches for breast cancer diagnosis

**DOI:** 10.3389/fmed.2024.1450103

**Published:** 2024-09-30

**Authors:** Faseela Abdullakutty, Younes Akbari, Somaya Al-Maadeed, Ahmed Bouridane, Iman M. Talaat, Rifat Hamoudi

**Affiliations:** ^1^Department of Computer Science and Engineering, Qatar University, Doha, Qatar; ^2^Computer Engineering Department, College of Computing and Informatics, University of Sharjah, Sharjah, United Arab Emirates; ^3^Clinical Sciences Department, College of Medicine, University of Sharjah, Sharjah, United Arab Emirates; ^4^Research Institute for Medical and Health Sciences, University of Sharjah, Sharjah, United Arab Emirates

**Keywords:** breast cancer detection, histopathology, multi-modality, XAI, machine learning and AI

## Abstract

Precision and timeliness in breast cancer detection are paramount for improving patient outcomes. Traditional diagnostic methods have predominantly relied on unimodal approaches, but recent advancements in medical data analytics have enabled the integration of diverse data sources beyond conventional imaging techniques. This review critically examines the transformative potential of integrating histopathology images with genomic data, clinical records, and patient histories to enhance diagnostic accuracy and comprehensiveness in multi-modal diagnostic techniques. It explores early, intermediate, and late fusion methods, as well as advanced deep multimodal fusion techniques, including encoder-decoder architectures, attention-based mechanisms, and graph neural networks. An overview of recent advancements in multimodal tasks such as Visual Question Answering (VQA), report generation, semantic segmentation, and cross-modal retrieval is provided, highlighting the utilization of generative AI and visual language models. Additionally, the review delves into the role of Explainable Artificial Intelligence (XAI) in elucidating the decision-making processes of sophisticated diagnostic algorithms, emphasizing the critical need for transparency and interpretability. By showcasing the importance of explainability, we demonstrate how XAI methods, including Grad-CAM, SHAP, LIME, trainable attention, and image captioning, enhance diagnostic precision, strengthen clinician confidence, and foster patient engagement. The review also discusses the latest XAI developments, such as X-VARs, LeGrad, LangXAI, LVLM-Interpret, and ex-ILP, to demonstrate their potential utility in multimodal breast cancer detection, while identifying key research gaps and proposing future directions for advancing the field.

## 1 Introduction

Breast cancer remains one of the leading causes of mortality worldwide, highlighting the critical need for accurate and timely diagnosis to improve patient outcomes. Historically, diagnostic methodologies have predominantly relied on unimodal approaches, which focus on a single type of data, such as imaging alone. While these methods have provided foundational insights, they are constrained by significant limitations. For example, unimodal approaches often suffer from reduced accuracy at higher magnifications, sensitivity to data imbalance, and limited generalizability across different datasets or conditions (1, 2).

**Figure 1:**
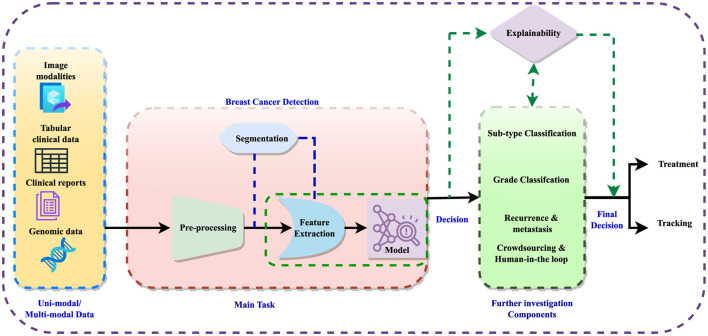
A generic representation of breast cancer diagnosis.

The detection process involves data preprocessing, feature extraction, and sometimes image segmentation to improve feature learning. Subsequently, detection models are employed to diagnose the disease, followed by further analyses such as subtype classification, grading, and prediction of recurrence or metastases. The integration of crowdsourcing and human-in-the-loop methodologies refines these analyses, enabling informed decisions regarding treatment and monitoring. Figure 1 illustrates the general workflow for breast cancer diagnosis within a multi-modal context, incorporating elements of explainability. Explainable AI (XAI) techniques are crucial in this context, as they aim to clarify the opaque nature of complex algorithms, explain the reasoning behind diagnostic decisions, and improve the interpretation of diagnostic results. Explainability not only enhances clinician confidence in decision support systems but also facilitates patient understanding and engagement, fostering informed decisions and personalized treatment plans.

The evolution of multi-modal diagnostic techniques marks a significant shift in the field of breast cancer detection and classification, emphasizing the integration of diverse data sources beyond traditional imaging. In addition to conventional imaging modalities such as mammography, magnetic resonance imaging, ultrasound, and positron emission tomography (PET), multi-modal approaches leverage a wide variety of non-image data types including genetic markers, proteomic profiles, clinical parameters, and patient demographics (3–5). By harnessing the complementary insights gleaned from these diverse data modalities, multi-modal techniques offer a multifaceted understanding of breast cancer biology and pathology, transcending the limitations of unimodal approaches.

**Figure 2:**
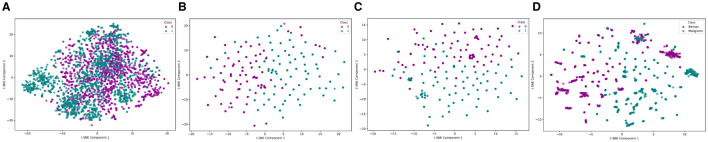
Feature space distribution of unimodal (VGG, BERT and Tabular) and multimodal (VGG + BERT + Tabular) data. **(A)** VGG, **(B)** BERT, **(C)** Tabular data, and **(D)** VGG + BERT + Tabular. In the unimodal plots, the labels Class 0 and Class 1 correspond to the Benign and Malignant classes, respectively.

The impact of incorporating multiple modalities can be demonstrated by comparing the feature space under unimodal and multimodal conditions. The comparative visualization of feature space distribution highlights the significant advantages of multimodal methods over unimodal approaches using the multimodal EMR dataset (6) in breast cancer diagnosis, as shown in Figure 2. Unimodal methods, as illustrated by the VGG-16 (Figure 2A), Bidirectional Encoder Representations from Transformers (BERT; Figure 2B), and tabular data (Figure 2C), exhibit limitations such as reduced accuracy, sensitivity to data imbalance, and poor generalizability across different datasets. These methods often fail to capture the complete picture due to their reliance on a single data type, leading to less distinct clustering and potential loss of critical discriminative features at higher magnifications. In contrast, the multi-modal approach, which integrates image, text, and tabular data, demonstrates superior clustering and separation of data points, reflecting enhanced diagnostic accuracy and robustness (5). This integration leverages complementary information from diverse data sources, providing a holistic view of breast cancer pathology, improving generalizability, and reducing the risk of overfitting. Consequently, multi-modal methods offer a more comprehensive and reliable diagnostic tool, addressing the inherent constraints of unimodal approaches (7). Figure 2, visually underscores these points by showing clearer data separation and clustering in the multi-modal plot compared to the unimodal ones.

Furthermore, alongside the integration of multimodal data, the necessity for explainability in breast cancer diagnosis emerges as a pivotal consideration. XAI is a technique that aims to clarify the opaque nature of complex algorithms, explaining the reasoning behind diagnostic decisions and improving the interpretation of diagnostic results (4). Explainability in breast cancer diagnosis not only enhances clinician confidence in decision support systems but also facilitates patient understanding and engagement, fostering informed decisions and facilitating personalized treatment plans.

Based on the above, it is important to focus on multimodal analysis in the medical field, especially in the field of breast cancer. Therefore, a comprehensive overview in this field can help researchers and industry to find frontiers and future directions and to develop and implement improved methods. Table 1 presents recent reviews on breast cancer diagnosis across various contexts. However, these reviews often overlook multi-modality and explainability, treating them as future research directions rather than discussing existing methods. Additionally, there is a lack of focus on histopathology and frameworks that combine histopathology with non-image data for breast cancer detection.

**Table 1:** Latest reviews on breast cancer diagnosis in various contexts.

**References**	**Main discussion**	**Datasets**	**Multi modality**	**XAI**
Abo-El-Rejalet al. (3)	Segmentation	×	×	×
Bai et al. (8)	Explainability	×	×	✓
Brodhead et al. (9)	Imaging characteristics	×	×	×
Hussain et al. (4)	Breast cancer risk prediction	×	✓	✓
Luo et al. (10)	Breast cancer imaging	×	✓	×
Rautela et al. (11)	Computational techniques for breast cancer	×	✓	×
Singh et al. (12)	Breast cancer screening and detection using artificial intelligence and radiomics	×	✓	×
Thakur et al. (13)	Identification and of breast cancer through medical image modalities	✓	✓	×

In light of these observations, this review addresses multi-modal datasets, including histopathology and other non-image data, and explores multi-modal techniques utilizing these datasets. It examines explainable multi-modal methods in histopathology-based breast cancer diagnosis, providing a comprehensive overview of the evolving field. Advances in medical data analytics now underscore the importance of these integrated methodologies, highlighting the fusion of histopathology images with non-image data. By integrating multi-modality and explainability, this review aims to contribute to the strategic direction of breast cancer diagnosis and treatment, ultimately enhancing diagnostic accuracy, clinician confidence, and patient outcomes. By showcasing how these combined approaches provide a more holistic and detailed perspective on breast cancer, we emphasize the critical role of multi-modal techniques in advancing the field and improving both diagnostic and therapeutic strategies.

The major contributions of this article are:

- A detailed investigation of multi-modal datasets, including those that incorporate histopathology and non-image data, which are frequently overlooked in existing literature.
- A discussion on multi-modal techniques that utilize the aforementioned datasets, offering insights into their application and effectiveness in breast cancer diagnosis.
- An investigation of explainable multi-modal methods specifically within the context of histopathology-based breast cancer diagnosis, addressing a critical gap in current research.
- Identification research gaps in multi-modality and explainability, identifying key areas for future study and contributing to the strategic direction of the field.

## 2 Breast cancer diagnosis: an overview

The diagnosis of breast cancer (14) involves a number of tasks, utilizing both image and non-image data. Using Machine learning (ML) algorithms, these data can be analyzed to identify potentially suspicious areas or anomalies that may indicate the presence of tumors. These advanced techniques (15) offer a more efficient and potentially more accurate method for detecting early signs of breast cancer, providing valuable insights for healthcare professionals in their diagnostic process.

Malignancy classification (16) is the process of determining whether detected abnormalities are malignant, indicating cancer, or benign, meaning they are non-cancerous. This step is vital for guiding the subsequent treatment plan. Machine learning models can assist in this classification by analyzing features derived from imaging data, including characteristics like shape, texture, and intensity. By training these models on large datasets, they can provide predictions on the probability that an abnormality is cancerous, aiding healthcare professionals in making informed decisions regarding patient care (17).

Subtype classification is a crucial process in understanding breast cancer, as it encompasses a spectrum of diseases, each with unique traits and outcomes (18). This step involves dividing breast cancer cases into specific subtypes like hormone receptor-positive, HER2-positive, or triple-negative breast cancer, which are known to have varying responses to treatments and differing prognoses. By categorizing cases into these subtypes, medical professionals can tailor treatment plans more effectively (19). Machine learning models play a role in this by analyzing genomic data, gene expression profiles, and clinical information to predict the subtype, facilitating personalized and targeted therapeutic approaches.

Image segmentation (15) involves dividing an image into cell segmentation and distinct segments or regions of interest. Within the realm of breast cancer diagnosis, segmentation helps to demarcate the boundaries of tumors or suspicious lesions in breast imaging data (43). This process is critical for precisely measuring tumor size and shape, and it lays the groundwork for further analyses, including tumor volume estimation or extracting quantitative features. Machine learning algorithms, especially deep learning models like convolutional neural networks (CNNs), have demonstrated strong capabilities in automatically segmenting breast lesions from medical images (44), offering a powerful tool to enhance the accuracy and efficiency of breast cancer diagnosis.

Predicting cancer recurrence and metastasis (45) is a crucial aspect of breast cancer management, extending beyond initial diagnosis and treatment. This task involves assessing the risk of the cancer returning or spreading to other parts of the body. Machine learning models can combine multiple types of data-such as imaging, genomic information, clinical variables (like patient demographics and medical history), and treatment records-to estimate the likelihood of recurrence or metastasis (37). These predictions are valuable for clinicians, allowing them to customize follow-up care and create personalized treatment plans for breast cancer patients, ultimately enhancing patient outcomes and reducing the risk of adverse events. It should be noted that the tasks should be combined and integrated to have an accurate system. For example, cancer detection for subtype classification should use the tasks of cancer segmentation and grading tasks and this process can improve the task of subtype classification (46).

Table 2 presents a summary of recent research advancements in breast cancer diagnosis across various tasks. A significant observation is the predominance of unimodal approaches in current methodologies. While some existing multimodal methods incorporate different types of imaging, such as ultrasound and mammography, the integration of image data with non-image data remains significantly underexplored. In particular, the fusion of histopathology images with non-image data, including textual and clinical information, represents a largely untapped area. The potential benefits of this integration are substantial. By combining histopathology imaging with comprehensive clinical and textual data, and leveraging advanced machine learning techniques, there is a strong potential to enhance the accuracy and efficiency of breast cancer diagnosis, prognosis, and treatment planning. This holistic approach could lead to significant advancements in personalized medicine and improved patient outcomes.

**Table 2:** Recent research in breast cancer diagnosis including different tasks.

**Method**	**Dataset**	**Modality**	**Task**
Classifier-combined method (16)	Proprietary	MRI	Grade classification
DeepBreast CancerNet (20)	BUSI (21), Ultrasound Image dataset (22)	Ultrasound	Detection
DSCCN (18)	TCGA (23)	multi-omics	Sub-type classification
EMDCOC (24)	BreakHis (25) IR Thermal Images (26)	Histopathology, IR thermal images	Detection
Ensemble CNN (17)	Databiox (27)	Histopathology	Grade classification
Histogram K-means segmentation (28)	BreakHis (25)	Histopathology	Segmentation
Hybrid CNN (29)	Mini-DDSM (30), BUSI (21)	Mammogram, ultrasound images	Detection
Hybrid CNN-LSTM (31)	BreakHis (25)	Histopathology	Grade classification
KAMnet (32)	Proprietary	Ultrasound	Detection
moBRCA-net (19)	TCGA (23)	Multi-omics,	Sub-type classification
Multi-modal fusion (33)	TCGA (23)	WSI, gene expression	Detection
Optimized LSTM with U-net segmentation (34)	MIAS (35)	Mammogram	Segmentation
Prediction model for distant metastasis (36)	Proprietary	Clinical Data	Reccurence and metastatis
Recurrence prediction (37)	WPBC	Clinical data	Recurrence and metastasis
Semantic segmentation (38)	CBIS-DDSM (39), MIAS (35)	Mammogram	Segmentation
Unet3+ (40)	Proprietary	Ultrasound	Segmentation
Yolo-based model (41)	CBIS-DDSM (39), Inbreast (42), Proprietary	Mammogram	Detection

## 3 Datasets

**Figure 3:**
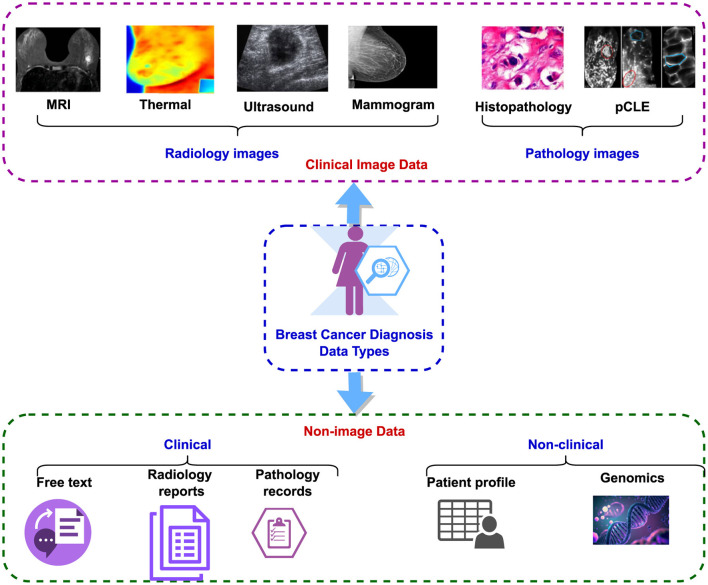
Types of breast cancer diagnosis data.

The dataset used for breast cancer diagnosis encompasses both clinical image data and non-image data (47), as illustrated in Figure 3. The clinical image data comprise radiology and pathology images. Radiology images encompass modalities such as MRI, CT, thermal imaging, mammograms, and ultrasound, while pathology images include histopathology and pCLE (5). The non-image data can be subdivided into clinical and non-clinical categories. Clinical data encompass radiology reports, pathology reports, including laboratory results, and narrative descriptions of patient status. Non-clinical data comprise patient profiles containing demographic information, patient history, age, other non-clinical details, and genomic data (48).

Additionally, non-image data are further classified into structured and unstructured categories. Radiology reports and narrative descriptions of patient status fall under unstructured data, while recorded pathology reports and patient profiles are considered structured data (49). Despite the abundance of both image and non-image datasets related to breast cancer detection, this paper focuses specifically on histopathology-based datasets, examining them in a multi-modal context. Table 3, lists the existing public datasets in breast cancer detection, based on histopathology. It is evident from the table that the number of multi-modal datasets is much less compared to the unimodal datasets. Also, the sample size is low in most of these datasets.

**Table 3:** Multi-modal datasets public for breast cancer diagnosis featuring histopathology images.

**Dataset**	**Year**	**Size**	**Modalities**
Post-NAT-BRCA (52)	2019	96	WSI, clinical data
CPTAC-BRCA (53)	2020	642	WSI, clinical, proteomic, genomic data
Pathological EMR (54)	2021		WSI, patient profile
BCNB (55)	2022	1,058	Clinical data
IMPRESS (56)	2023	126	WSI, clinical data
GTEx-Breast dataset (57)	2023	894	WSI, pathology notes
TCGA-BRCA dataset (23)	2023	1,098	WSI, gene expression, CNV

The landscape of breast cancer histopathology research is enriched by a diverse array of datasets, each offering unique features and clinical insights. Uni-modal datasets, such as BRACS (50) and BreCaHAD (51), focus on a single type of data. The BRACS dataset provides 547 Whole-Slide Images (WSIs) and 4,539 Regions Of Interest (ROIs), meticulously annotated by three board-certified pathologists. This dataset categorizes lesions into types such as Normal, Pathological Benign, Usual Ductal Hyperplasia, Flat Epithelial Atypia, Atypical Ductal Hyperplasia, Ductal Carcinoma in Situ, and Invasive Carcinoma. Similarly, the BreCaHAD dataset includes 162 histopathology images focusing on malignant cases, classified into mitosis, apoptosis, tumor nuclei, non-tumor nuclei, tubule, and non-tubule, thus facilitating comprehensive analyses and validation of diagnostic methods.

In contrast, multi-modal datasets integrate various data types to provide a more comprehensive view of breast cancer pathology. The TCGA-BRCA (23) dataset, for instance, combines gene expression data, copy number variations (CNVs), and pathological images from 1,098 breast cancer patients. This multi-dimensional approach allows for a deeper understanding of the molecular and histological characteristics of breast cancer. Similarly, the IMPRESS dataset includes Hematoxylin and Eosin (H&E) and immunohistochemistry (IHC) stained WSIs from 126 patients, along with clinical data and biomarker annotations. The Post-NAT-BRCA38 dataset (52) offers 96 WSIs along with detailed clinical information, including estrogen receptor (ER) status, progesterone receptor (PR) status, and human epidermal growth factor receptor 2 (HER2) status. These multi-modal datasets enable researchers to explore the interplay between genetic, molecular, and histological data, driving advancements in personalized breast cancer diagnosis and treatment.

The IMPRESS dataset (56) consists of 126 breast H&E WSIs from 62 female patients with HER2-positive breast cancer and 64 female patients with triple-negative breast cancer, all of whom underwent neoadjuvant chemotherapy followed by surgical excision. It includes immunohistochemistry (IHC) stained WSIs of the same slides, along with corresponding scores. All slides were scanned using a Hamamatsu scanner at 20 × magnification. The dataset also provides clinical data for both patient groups, including age, tumor size, and annotations for biomarkers such as PD-L1, CD-8, and CD-163. The GTEx-Breast dataset (57) is part of the Genotype-Tissue Expression (GTEx) project, which offers gene expression data across 44 human tissues. It includes 894 breast tissue histology images, comprising 306 WSIs of female breast tissue and 588 WSIs of male breast tissue, collected from the central subareolar region of the right breast at various centers in the United States. The images are accompanied by brief pathology notes and an annotation file with detailed sample information.

The CPTAC-BRCA dataset (53), from the Clinical Proteomic Tumor Analysis Consortium, includes 642 WSIs from 134 patients with breast invasive carcinoma, scanned at 20 × magnification. The images are available in two resolutions: 0.25 and 0.5 *mu*m/pixel. The dataset is accompanied by comprehensive clinical, proteomic, and genomic data. The BCNB dataset (55), or Early Breast Cancer Core-Needle Biopsy WSI Dataset, is the only publicly available collection of breast histopathology WSIs from Asia. It contains 1,058 WSIs from 1,058 breast cancer patients in China, scanned with an Iscan Coreo pathological scanner. Tumor regions in each image are annotated by two pathologists. The dataset also includes extensive clinical data such as patient age, tumor size, histological and molecular subtypes, number of lymph node metastases, and HER2, ER, and PR status.

A multi-modal evaluation should require the selection of datasets that include comprehensive and relevant data across various modalities, including imaging, clinical records, and genomic data. Using the selected datasets would allow a robust and comprehensive assessment of the multimodal approach's effectiveness and applicability. As an inclusion criterion, it would be desirable to identify datasets that included all required modalities and met high-quality standards, such as high-resolution imaging, complete and standardized clinical information, and accurate genomic sequencing. Additionally, datasets should be selected based on their clinical relevance, ensuring that they reflect a diverse range of patient demographics (e.g., age, gender, ethnicity) and a variety of cancer subtypes, both of which are crucial for mimicking real-world clinical conditions. An exclusion criteria should be used to exclude datasets that did not meet these standards, including incomplete data modalities, low-quality data (e.g., low-resolution images or missing clinical information) and samples that did not represent a broad range of patient groups and cancer types. Using this rigorous selection process ensures that the datasets used are representative of real-world clinical scenarios, thus making the study more generalizable and relevant. For example, structured EMR dataset (54) was selected for its comprehensive imaging and clinical data across a diverse patient cohort, while TCGA-BRCA (23) was chosen for its detailed genomic data and its inclusion of multiple cancer subtypes, mirroring the heterogeneity observed in clinical practice. By clearly defining these inclusion and exclusion criteria, we aimed to ensure that the selected datasets are both comprehensive and high-quality, as well as representative of diverse real-world clinical environments, thereby ensuring the robustness and validity of the multi-modal approach under evaluation in this study.

## 4 Histopathology-driven breast cancer diagnosis

Histopathology-driven breast cancer detection leverages microscopic examination of tissue samples to diagnose and understand the progression of breast cancer. This approach involves the detailed analysis of histological images, where pathologists identify abnormal cellular structures indicative of malignancy. In recent years, advancements in artificial intelligence (AI) and machine learning have significantly enhanced histopathology analysis, enabling more accurate and efficient detection of cancerous cells. AI models, particularly those employing deep learning techniques, can process large volumes of high-resolution images, extracting critical features that might be overlooked by human eyes. These models assist in classifying tissue samples, predicting cancer subtypes, and providing prognostic information, thus playing a crucial role in personalized treatment planning. The integration of AI in histopathology not only improves diagnostic accuracy but also addresses challenges such as inter-observer variability and the increasing demand for pathological assessments, ultimately contributing to better clinical outcomes for breast cancer patients.

### 4.1 Uni-modal techniques

Histopathology-based uni-modal breast cancer detection remains a critical medical approach, utilizing microscopic examination of tissue samples to identify cellular abnormalities. Numerous methods have been developed leveraging histopathology images for this purpose. This section provides a comprehensive analysis of recent uni-modal techniques in histopathology-based breast cancer detection.

#### 4.1.1 A comprehensive review on uni-modal techniques

Gan and Subasi (58) proposed a method for low-magnification histopathology grading improved data learnability by using data augmentation and the CovXNet model. This improved generalization capacity, regression optimization, and feature purification. The CovXNet model captured features at multiple observation levels, achieving the highest classification accuracy of 92.13% for the Breast Histopathology Images dataset. However, GAN-generated patches did not improve validation accuracy or class distinction. Another method utilized deep learning on the IDC dataset, revealing that VGG16 and MobileNet architectures achieved nearly 92% accuracy in detecting breast cancer (59). In Zhang et al. (60) a novel classification framework for analyzing whole slide breast histopathology images (WSI) was introduced. The approach involved patch-based classification, tumor region segmentation and location, and WSI-based classification. Techniques utilized included Cycle-GAN for image color normalization, a fused model combining DPN68 and Swin-Transformer for enhanced patch-based classification accuracy, and SVM for the final WSI-based classification. This method effectively addressed the challenge of processing large WSIs directly and provided a visual heatmap to facilitate better tumor diagnosis. Solorzano et al. (61) compared a single CNN model to an ensemble of ten InceptionV3 models to detect invasive breast cancer (IC) in histopathology images. The ensemble model outperformed the single CNN model in accuracy on the tile level in 89% of all WSIs in the test set. The overall accuracy was 0.92 for the ensemble model in the internal test set and 0.87 for the TCGA dataset. However, the study acknowledged the limitation of having 587 WSI in the internal datasets, which may affect the generalizability of the findings. Future work could explore the explainability of ensemble models and evaluate the impact of IC detection on downstream analysis tasks.

A deep learning technique and multiple instance learning (MIL) method for classifying histopathology breast cancer images was presented in Maleki et al. (62). It utilized pre-trained models and an extreme gradient boosting classifier to improve accuracy. The method exhibited high accuracy across various magnification levels and demonstrated robustness across different resolutions. However, its accuracy decreased at higher magnification levels due to the loss of discriminative features. A rank-based ensemble method that utilized the Gamma function to classify breast histopathology images was presented in Majumdar et al. (63). This method outperformed state-of-the-art techniques, achieving classification accuracies of 99.16%, 98.24%, 98.67%, and 96.16% across different magnifications on the BreakHis dataset and 96.95% on the ICIAR-2018 dataset. Despite its promising results, the method had limitations, such as its application to a single data modality and the need for further validation across other data modalities to ensure its generalization ability.

Using color normalization and nucleus extraction techniques, the method (64) evaluated H&E and fluorescent staining technologies for the detection of breast cancer tumors. An AI model was developed for segmenting H&E-stained images, enabling cross-staining recognition between bright-field and dark-field images. This approach maintained a high level of precision in tumor feature recognition across different staining methods with high accuracy rates. However, the method acknowledged that fluorescent signals fade over time, making their use less common in daily practice. Additionally, the high data requirement for developing deep learning models posed a significant entry barrier for special stains such as fluorescent stains. Hist2RNA (65), a deep learning-based method was designed to predict gene expression from digital images of stained tissue samples, aiming to enhance breast cancer diagnosis and treatment by enabling personalized therapies. It proved to be more efficient and computationally less demanding than traditional molecular tests and could identify breast cancer subtypes, thereby facilitating targeted treatment strategies. However, its generalizability was limited due to its focus on LumA and LumB subtypes, and it potentially introduced extra noise in subtype classification due to tissue heterogeneity and staining variability. Additionally, there was a lack of rigorous external validation because of the absence of molecular information in the TMA dataset used. Future directions included expanding validation on a more diverse dataset, developing robust algorithms for image analysis and validation, and integrating Hist2RNA into clinical practice.

The AOADL-HBCC technique (66) employed an arithmetic optimization algorithm (AOA) and a SqueezeNet model for feature extraction from histopathology breast cancer images. It included preprocessing steps such as noise removal and contrast enhancement to improve image quality. The method utilized a deep belief network classifier with an Adamax hyperparameter optimizer for classification. The AOADL-HBCC method demonstrated superior performance in breast cancer classification, with increased training and validation accuracy and minimal training and validation loss. Additionally, the method showed proficiency in classifying different classes in the test database, as evidenced by a brief ROC study. A Convolutional Neural Network (CNN)-based binary classification method (67) was used to diagnose cancer from histopathology images. The CNN architecture extracted features and classified images with high accuracy. The model achieved a prediction accuracy of up to 99.86%, in improving cancer diagnosis. However, the model's performance varied depending on the quality and diversity of the input data. To improve the detection performance of breast cancer histopathology images, the method (68) combined dilated convolution, ResNet, and AlexNet. It introduced a Composite Dilated Backbone Network (CDBN), which integrated multiple identical backbones into a single robust network. The CDBN improved mean Average Precision (mAP) by 1.5%–3.0% on the BreakHis dataset and enhanced instance segmentation, elevating the baseline detector cascade mask R-CNN to an mAP of 53.3. The proposed detector did not require pretraining, thereby simplifying integration into existing workflows. However, the method required significant computational resources and struggled with extremely varied or low-quality histopathology images.

Using a multistage approach, Mahmood et al. (69) detected mitotic cells in breast cancer histopathology images through the use of Faster region convolutional neural networks (Faster R-CNNs) for initial detection, Deep Convolutional Neural Networks (Deep CNNs) for feature extraction, post-processing for false-positive reduction, and machine learning. These methods collectively contributed to improving the accuracy and reliability of mitotic cell detection in breast cancer diagnosis. However, the approach had several limitations, including limited data availability for training deep learning models, high computational costs, and challenges in generalization capability. Despite employing data augmentation techniques like flipping and translation to mitigate data scarcity, the inherent lack of data remained a significant constraint. To classify breast cancer histopathology images into non-carcinoma and carcinoma classes, an ensemble of deep learning models, specifically VGG16 and VGG19, was utilized in Hameed et al. (70). The ensemble approach demonstrated a high sensitivity of 97.73% for the carcinoma class and an overall accuracy of 95.29%, indicating a significant improvement in accurately classifying the complex nature of breast cancer histopathology images. The model also achieved an F1 score of 95.29%, showcasing balanced precision and recall, which is crucial for medical diagnostic systems. However, the approach had limitations, including the use of a small dataset, which could restrict the model's generalizability to a wider range of histopathology images not represented in the training set. Additionally, the focus on only two classes might not capture the full spectrum of breast cancer histopathology, potentially limiting its applicability to more nuanced diagnostic scenarios. A modified Inception_V3 and Inception_ResNet_V2 architecture was used in Xie et al. (71) to extract high-level abstract features from histopathology images of breast cancer. These architectures were adjusted for binary and multi-class classification issues. The model was adapted and balanced by manipulating images to mitigate imbalanced data. The results showed superior classification accuracy compared to traditional methods, with the Inception_ResNet_V2 architecture proving to be the most effective. The features extracted were used for unsupervized analysis, demonstrating better clustering results with a newly constructed autoencoder network. However, the study's reliance on deep learning models required substantial computational resources, which may not have been accessible in all research or clinical settings.

#### 4.1.2 Uni-modal techniques: a critical analysis

Unimodal methods, which rely on single types of data or features, demonstrated significant limitations in breast cancer histopathology, particularly when applied to higher magnification levels such as 400 × due to the potential loss of discriminative power of features (62). This reduction in accuracy can lead to biased models, thereby affecting the overall performance. Additionally, unimodal approaches are highly sensitive to data imbalance, struggle with unbalanced class distributions, and often exhibit limited generalizability across different datasets or conditions, particularly in biomedical applications where sample variability is common. Furthermore, these methods are prone to overfitting, especially when dealing with complex or high-dimensional data, underscoring the need for multi-modal approaches that leverage various data types and analytical methods to enhance robustness and accuracy.

Relying on a single data modality, such as histology images alone, presents inherent constraints, including a limited perspective and restricted generalization ability (63). Unimodal methods may miss complementary information from other modalities, thereby limiting the model's understanding and representation of the problem. In contrast, multi-modal methods integrate multiple data types, enhancing the model's robustness and adaptability through comprehensive analysis, improved feature representation, and increased robustness to noise and variability. By incorporating data from multiple sources, multi-modal approaches can uncover patterns not visible through unimodal methods, thereby offering a more holistic view of cancerous tissues and improving diagnostic confidence (72).

Traditional unimodal histopathology methods, despite their long-standing use, face significant limitations compared to the potential benefits of integrating artificial intelligence (AI) (73). These limitations include high integration costs, regulatory hurdles, substantial initial investments, and data protection challenges. The transition to AI-enhanced processes is financially and logistically challenging, as AI applications in clinical settings face stringent regulatory approvals and require substantial computational resources. This shift is further complicated by the need for significant redundancy and backup measures to ensure patient data protection.

In contrast, multi-modal methods in breast cancer histopathology offer enhanced detection capabilities by identifying a wider range of biomarkers and cellular activities, providing a detailed understanding of tumor cells. These methods reduce the likelihood of misdiagnosis, particularly in complex cases where traditional methods may be insufficient. Multi-modal approaches enable comprehensive analysis of multiple factors, such as biomarker presence and cell spatial distribution, leading to a nuanced understanding of the disease. Although initially more costly, multi-modal methods ultimately save resources by reducing the need for repeat tests and follow-up procedures, thereby streamlining the diagnostic process (67).

The limitations of unimodal methods, such as their focus on specific breast cancer subtypes and the introduction of noise due to tissue heterogeneity and staining variability, highlight the need for multi-modal methods (65). By integrating genetic, imaging, and clinical data, multi-modal approaches enhance generalizability and reduce noise, leading to more accurate and reliable predictions. These methods also enable comprehensive validation across diverse datasets, bolstering the robustness and reliability of predictive models.

The advantages of using multi-modal methods over unimodal methods for detecting mitotic cells in breast cancer histopathology images are well-documented. Multi-modal approaches offer enhanced discrimination abilities, improved accuracy and reliability, noise reduction, and better generalization capability. By fusing data from multiple modalities, these methods provide superior discrimination abilities crucial for high-accuracy applications like medical diagnosis and are more effective for real-time clinical applications (69).

In summary, while unimodal methods have provided foundational insights into breast cancer histopathology, their limitations underscore the need for multi-modal approaches that leverage the strengths of various data types, thereby promising more accurate and clinically relevant outcomes.

### 4.2 Multi-modal techniques

Multi-modal techniques are essential in histopathology-based breast cancer detection for improved diagnostic accuracy, comprehensive insights, and patient outcomes. These techniques combine various data modalities, such as histopathology images, molecular profiles, and clinical data, to differentiate between cancer subtypes, assess tumor heterogeneity, and predict treatment responses. Advanced imaging and computational tools, like machine learning and artificial intelligence, have revolutionized histopathology data analysis, automating detection and classification, extracting complex patterns, and providing decision support to pathologists. These techniques facilitate a deeper understanding of breast cancer mechanisms, leading to the discovery of new therapeutic targets and biomarkers.

#### 4.2.1 An analysis of current existing multi-modal techniques

**Figure 4:**
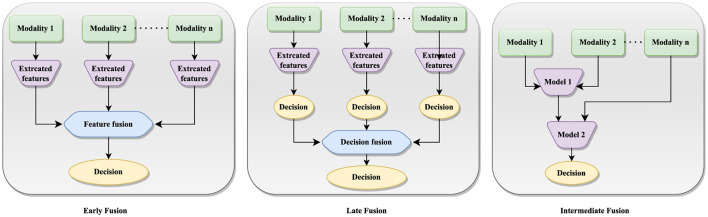
Graphical representation of early, late and intermediate fusion implementation.

The multi-modal fusion can be categorized as stage-based and method-based techniques. Stage-based fusion strategies can be further categorized into early, late, and intermediate fusion approaches (74), each offering unique advantages in breast cancer detection. Figure 4 illustrates the implementation of early, late and intermediate fusion techniques. This approach is particularly beneficial when uni-modal data are noisy or incomplete, as integrating redundant information from other modalities can improve the robustness and precision of predictions.

Multi-modal fusion approaches (75) include encoder-decoder methods, which combine feature extraction, fusion, and decision-making processes into a single model, making them efficient in tasks like video captioning and object detection. Attention mechanism methods use mechanisms like co-attention and cross-attention to enhance each modality with information from other modalities, allowing the model to fuse features and learn interdependencies among them. Graph Neural Network methods use GNN to capture long-range dependencies among different modalities, categorizing tasks into different classes based on data types. Generative Neural Network methods include models like VAE-based adversarial frameworks, which reduce distance differences between unimodal representations and are crucial for tasks like text-conditional image generation and image style transfer. Constrained-based methods involve innovative approaches like channel-exchanging-networks, which dynamically exchange channels in different modal sub-networks based on individual channel importance, but are limited to homogeneous data.

Multi-modal techniques offer a more accurate, comprehensive, and personalized approach to breast cancer diagnosis and treatment, but they face challenges such as data standardization, computational resources management, and interdisciplinary collaboration. Future advancements in technology and computational methods are expected to address these challenges, making multimodal techniques more effective and widely adopted in clinical practice. However, challenges persist, such as the richness of feature representation (54) in images and the inadequacy of information fusion, which can lead to the loss of high-dimensional information and partially missing data in real-world scenarios. Each modality within multimodal data possesses distinct characteristics, adding to the complexity of heterogeneous data and further complicating multimodal fusion methods.

The integration of multi-modal approaches in breast cancer diagnosis, including histopathology and non-image modalities, improves diagnostic accuracy, provides a comprehensive understanding of the disease, improves personalized treatment planning, facilitates early detection and timely intervention, potentially improving patient outcomes, and promotes interdisciplinary collaboration among specialists. This approach reduces the likelihood of misdiagnosis, provides a more comprehensive understanding of tumor biology and patient health, and facilitates early detection and timely intervention, ultimately advancing clinical research. Table 4 shows recent multi-modal research in breast cancer diagnosis.

**Table 4:** Existing research in multi-modal breast cancer diagnosis.

**References**	**Datasets**	**Fusion strategy**	**Modality**
Sun et al. (78)	METABRIC	Late fusion	Clinical data, Gene expression
Tong et al. (79)	TCGA-BRCA	Encoder-decoder method	Gene expressions, CNV
Arya and Saha (80)	METABRIC, TCGA-BRCA	Early fusion	Clinical data, Gene expression
Subramanian et al. (81)	TCGA-BRCA	Early fusion	Histopathology images, Clinical data
Liu et al. (82)	TCGA-BRCA	Late fusion	Histopathology images, Gene expressions
Howard et al. (83)	TCGA-BRCA	Late fusion	Histopathology images, Gene expressions
Arya and Saha (84)	METABRIC, TCGA-BRCA	Encoder-decoder method	Clinical data, Gene expression
Arya and Saha (85)	METABRIC	Early fusion	Clinical data, Gene expression
Furtney et al. (86)	TCGA-BRCA	Graph-neural network method	Histopathology images, Clinical data, Gene expressions, Radiological data
Rani et al. (87)	TCGA-BRCA	Early fusion	Histopathology images, Gene expressions
Kayikci et al. (88)	METABRIC	Attention-based	Clinical data, Gene expression
Arya et al.(89)	TCGA-BRCA	Early fusion	Clinical data, Gene expression
Mondol et al. (90)	TCGA-BRCA	Attention-based	Histopathology images, Clinical data, Gene expressions
Huang et al. (91)	TCGA-BRCA, GMUCH-BRCA	Early fusion	Histopathology images, Clinical data
Li and Nabavi (92)	TCGA-BRCA	Graph-neural network method	Gene expressions, CNV

The integration of heterogeneous data, particularly maintaining the integrity of high-dimensional image information, has been a challenging aspect of data fusion. Yan et al. (54) developed a multi-modal fusion technique to improve the dimensionality of structured data in histopathology whole slide images (WSI). They used VGG-16 for image feature extraction and a denoising autoencoder to enhance clinical data. These features were combined into fully connected layers for classification, distinguishing between benign and malignant cases using a pathological electronic medical record dataset. Yellapragada et al. (76) proposed PathLDM, a text-conditioned Latent Diffusion Model, to enhance histopathology image generation by integrating contextual information from pathology text reports. The model achieved a leading Fréchet Inception Distance (FID) score of 7.64 on the TCGA-BRCA dataset, outperforming other models in generating high-quality, text-conditioned histopathology images.

It is essential to implement strategies that ensure a balanced integration of all modalities in order to avoid potential biases during data fusion. This is particularly important when certain modalities are over-represented or when the quality of the data varies significantly (77). To begin with, bias sources, such as over-representation of certain modalities or inconsistencies in data quality, should be identified, as these can disproportionately influence model outcomes. In order to mitigate these biases, modalities can be normalized and standardized in order to ensure that they contribute equally, and weighted fusion can be applied in order to balance their impact according to their reliability. Moreover, quality assessment and filtering can be used to manage variations in data quality, and data augmentation can be used to enhance the representation of underrepresented modalities. Furthermore, the effectiveness of these strategies should be evaluated through comparative analyses, cross-validation, and sensitivity analysis in order to minimize bias and enhance the model's generalizability and performance.

Ding et al. (93) developed a new method for mitosis detection in histopathology images using large vision-language models. They integrated image captioning and visual question-answering tasks with pre-trained models, incorporating metadata like tumor and scanner types. This approach improved prediction accuracy and outperformed baseline models. MI-Zero (94) is another multi-modal framework, which used contrastively aligned image and text models for zero-shot transfer on gigapixel histopathology whole slide images. The framework used multiple instance learning and a graph-based representation, resulting in improved cancer subtype classification accuracy and robustness. A bi-phase model (80) was developed to predict breast cancer prognosis using genomic information, histopathology images, and clinical details. The model was evaluated using METABRIC and TCGA-BRCA datasets. The fusion strategy involved feature extraction, concatenation, and random forest classifiers. This enhanced the model's predictive power, utilizing the strengths of each modality and improving the accuracy of breast cancer prognosis prediction.

A hybrid deep learning model (82) effectively predicted molecular subtypes of breast cancer by integrating gene expression data with pathological images. They used the TCGA-BRCA dataset, selected 831 samples, processed gene expression data, and analyzed pathological images in RGB color. Using data from The Cancer Genome Atlas, Howard et al. (83) used a deep learning model to predict recurrence assay results and risk in breast cancer patients. The model extracted tessellated image tiles from tumor regions and downscaled them through a convolutional neural network. The model incorporated digital histology and clinical risk factors, resulting in patient-level predictions that outperformed traditional nomograms, enhancing the accuracy of recurrence predictions.

Canonical Correlation Analysis (CCA) and its penalized variants (pCCA) were used for multi-modality fusion (81) in breast cancer prediction, combining histopathology and RNA-sequencing data from breast cancer patients in The Cancer Genome Atlas (TCGA). A two-stage prediction pipeline was proposed using pCCA embeddings for latent variable prediction, enhancing survival prediction in breast cancer patients. The model outperformed Principal Components Analysis (PCA) embeddings in survival prediction tasks. A deep learning approach was proposed for survival risk stratification in breast cancer, integrating histopathology imaging, genetic, and clinical data. The MaxViT model was used for image feature extraction, with self-attention mechanisms capturing intricate patient relationships (90). A dual cross-attention mechanism fused image features with genetic data to enhance predictive accuracy. The study used the TCGA-BRCA dataset, which included 249 whole-slide images and clinical variables like tumor grade, size, patient age, and lymph node status.

Sun et al. (78) developed a Multimodal Deep Neural Network (MDNNMD) for breast cancer prognosis prediction using the METABRIC dataset. The method, which combined gene expression profiles, CNA profiles, and clinical information from 1,980 breast cancer patients, outperformed single-dimensional methods like DNN-Clinical, DNN-Expr, and DNN-CNA, demonstrating the superior predictive power of integrating multi-dimensional data for prognostic assessments in breast cancer. Arya and Saha (84) developed the Generative Incomplete Multi-View Prediction Model (GIMPP) to address missing views in breast cancer prognosis prediction. The model used multi-view encoder networks and view-specific generative adversarial networks to learn shared latent space representations. Validated on the TCGA-BRCA and METABRIC datasets, it demonstrated superior performance compared to state-of-the-art approaches.

Huang et al. (91) proposed a multimodal Siamese model for breast cancer survival prediction, Siamese-RegNet, which integrates pathological images with clinical data. The model extracts survival-related features from image patches and captures correlations between different modalities. The model demonstrated enhanced survival prediction accuracy using the TCGA-BRCA and GMUCH-BRCA datasets. Another multi-modal method (86) employed a cross-entropy loss function to batch patient graphs for training and to update graph embeddings within a Graph Neural Network (GNN) framework. The dataset utilized was the Cancer Genome Atlas Breast Invasive Carcinoma (TCGA-BRCA), comprising clinical, genomic, and radiological data from 1,040 patients. The approach involved multimodal fusion using graph convolutional neural networks (GCNs), with the goal of improving the model's generalization capabilities and overall performance. This strategy highlighted the potential of integrating diverse data types to enhance predictive accuracy in breast cancer prognosis.

It is essential to employ a variety of strategies in order to mitigate overfitting concerns in the context of multi-modal data and deep learning models in order to validate their performance and mitigate this risk. Due to the complexity of integrating multiple data modalities and the inherent complexity of deep learning architectures, there is a risk of overfitting (95) Multiple techniques were employed to counteract this, including cross-validation, such as k-fold cross-validation, which provides an assessment of generalizability across different subsets of data. Furthermore, regularization techniques such as L1 and L2 regularization, dropouts, and early stopping were applied to prevent the model from becoming too complex and to enhance its generalizability. Additionally, external validation datasets were used to ensure that model performance was tested on unseen data, adding a further level of robustness to the model. A paired *t*-test and Wilcoxon signed-rank test were conducted to evaluate the significance of the performance differences between the multi-modal and unimodal approaches for validating model performance statistically. As a measure of the reliability and uncertainty of the results, confidence intervals were calculated for key performance metrics. This comprehensive approach to mitigating overfitting and demonstrating the robustness and effectiveness of multi-modal models is achieved through the integration of these strategies and statistical methods.

Multi-modal breast cancer detection requires a nuanced approach to evaluating AI models that takes into account the unique characteristics of medical datasets, such as class imbalance and the importance of minimizing false negatives. Accuracy is a commonly used metric to determine the proportion of instances that are correctly predicted (96). Nevertheless, relying solely on accuracy can be misleading, especially in cases where the majority class (e.g., benign cases) outnumbers the minority class (e.g., malignant cases). Such scenarios may lead to the appearance of accuracy for a model that primarily predicts the majority class, but fail to identify critical cases. For a more realistic assessment, additional metrics are required. In this context, recall (sensitivity) is critical as it serves as a measure of the model's ability to correctly identify all instances of breast cancer while ensuring there are no false negatives. It is essential to have a high recall rate in order to detect diseases early and treat them effectively. With an F1-score, a single metric that considers both false positives and false negatives, it is ideal for datasets with class imbalances, where false alarms and missed diagnoses can have a significant impact (97). In addition to measuring the model's ability to differentiate between cancerous and non-cancerous cases across various thresholds, AUC-ROC provides a comprehensive assessment of the model's diagnostic performance. In order to specifically address class imbalances, metric such as balance accuracy, precision-recall curves, and Matthews Correlation Coefficients (MCCs) have been used. By averaging recall across classes, balanced accuracy takes into account class prevalence in order to reduce bias toward the majority class. The precision-recall curves provide insight into the trade-offs between precision and recall, particularly when cancer detection (the minority class) is of primary importance. MCC provides a balanced measure that considers all aspects of a confusion matrix, providing a more informative evaluation for binary classifications when the data is imbalanced. By reporting these metrics comprehensively, a robust and balanced evaluation can be conducted of AI models in multi-modal breast cancer detection, improving their reliability and effectiveness across a variety of clinical scenarios, thereby contributing to improved patient outcomes (98).

In order to create a comprehensive and unbiased assessment of multi-modal vs. traditional unimodal approaches, it is essential to follow a systematic process. By selecting models specific to each data modality, such as text, images, and structured data, the unimodal baselines are first defined. Following this, appropriate datasets must be selected, ensuring that both multimodal and unimodal models can be trained and tested on the same data. A consistent preprocessing, feature engineering, and evaluation metric should be used throughout the entire training and evaluation process. Furthermore, statistical significance testing, such as paired *t*-tests and Wilcoxon signed-rank tests, should be conducted in order to determine if performance improvements are statistically significant between the multi-modal model and each unimodal baseline, and *p*-values should be provided to indicate if the improvements are statistically significant.

Table 5 presents the performance of various unimodal and multi-modal methods in breast cancer detection, highlighting the accuracy achieved by each approach as reported by the authors. The comparison is carried out on same dataset for unimodal and multimodal scenarios. Unimodal methods, which rely on a single type of data modality, have demonstrated varying levels of accuracy in breast cancer detection. Yan et al. (6) reported an accuracy of 83.6% using image data and 81.5% using clinical data. Arya et al. (80) explored multiple unimodal approaches including clinical data (80.2%), gene expression data (80.6%), and copy number data (74.8%). These results illustrate that while unimodal methods can achieve reasonably high accuracy, there are notable differences depending on the type of data used.

**Table 5:** Performance of multi-modal techniques in breast cancer detection.

**References**	**Unimodal**		**Multi-modal**	
	**Modality**	**Accuracy**	**Method**	**Accuracy**
Yan et al. (6)	Image	83.6	Hybrid deep learning	90.6
	Clinical	81.5		
Yan et al. (54)	Image	83.6	Richer fusion network	92.9
	Clinical	78.5		
Sun et al. (78)	Clinical	–	MDNNMD	82.6
	Gene expression	–		
	Copy number	–		
Arya and Saha (84)	Clinical	–	GIMPP	86.9
	Gene expression	–		
	Copy number	–		
Arya and Saha (85)	Clinical	80.2	Stacked RF	90.2
	Gene expression	80.6		
	Copy number	74.8		

In contrast, multi-modal approaches, which integrate multiple types of data, consistently outperformed unimodal methods. Yan et al. (6) reported a significant increase in accuracy to 90.6% with their hybrid deep learning approach combining image and clinical data. Sun et al. (78) utilized a multi-modal approach incorporating clinical, gene expression, and copy number data, achieving an accuracy of 82.6% with their MDNNMD method. Arya and Saha (84) reported an accuracy of 86.9% using the GIMPP method and 90.2% with the stacked RF approach (80), both combining multiple data modalities.

Based on the comparative data in Table 5, paired *t*-tests and Wilcoxon signed-rank tests were conducted on the uni-modal and multi-modal results reported in Yan et al. (6, 54) and Arya and Saha (85), which utilized the same dataset. The paired *t*-test for Yan et al. (6, 54) results shows a *p*-value of 0.0080, indicating a statistically significant difference (*p* < 0.05) and suggesting that the multi-modal approach significantly outperforms the unimodal methods, assuming normal distribution of differences. However, the Wilcoxon signed-rank test for the same data provides a *p*-value of 0.1250, exceeding the 0.05 threshold, indicating insufficient evidence to confirm this improvement without assuming normality. Similarly, tests on data from Arya and Saha (85) yield a paired *t*-test *p*-value of 0.0247, again suggesting a significant difference under the normality assumption. In contrast, the Wilcoxon signed-rank test results in a *p*-value of 0.2500, further supporting the lack of significance without normal distribution. These findings underscore the need to consider data distribution assumptions when evaluating the comparative performance of uni-modal and multi-modal approaches and suggest further research is needed for more conclusive evidence.

The comparative analysis between unimodal and multi-modal methods reveals a clear advantage of multi-modal approaches in breast cancer detection. As a result of their unique ability to leverage the strengths of diverse data types, they are able to produce enhanced feature representations, robustness, and generalizability (99). However, while the findings suggest significant potential for improving clinical outcomes, they also underscore the need for ongoing research and development to further refine these methodologies in order to ensure their applicability across a variety of clinical settings and breast cancer subtypes (87, 100).

A number of challenges are highlighted in this analysis, including the variability in model performance across different breast cancer subtypes and the potential for overfitting due to the complexity of multimodal models and the limited size of the datasets (101). Inconsistencies in the detection of rarer cancers, such as triple-negative breast cancer, suggest that the effectiveness of multi-modal methods is dependent upon how subtypes are represented within training datasets (100). It is therefore imperative that future research prioritize more balanced datasets and advanced data integration techniques in order to increase the robustness and generalizability of models.

An important factor in determining the effectiveness of multi-modal approaches is the quality and integration of the data modalities involved. Several factors may undermine the accuracy of a model, such as poor resolution imaging data or genomic data with significant noise. These factors emphasize the importance of rigorous data preprocessing (102). As a result, although multi-modal approaches generally outperformed unimodal approaches in most cases, there were instances where unimodal approaches yielded comparable results, particularly when the single modality data was of high quality and relevance. Hence, multi-modal approaches may not always be necessary or advantageous, depending on the specific clinical context. In order to develop advanced, reliable AI-driven diagnostic tools for breast cancer detection, a balanced and critical evaluation of these methods is necessary. Further validation and research will also be required, along with further validation and research (6, 101).

#### 4.2.2 Error analysis in multi-modal breast cancer detection

A thorough error analysis is critical in multi-modal breast cancer detection to understand the specific areas where AI models may fail and to enhance their overall performance. Given the complexity and heterogeneity of breast cancer, which includes multiple subtypes and varying data modalities (such as imaging, genomic profiles, and clinical records) (103), identifying the specific failure points of AI models is crucial for guiding future improvements and optimizing clinical outcomes.

One of the key aspects of error analysis involves examining how AI models perform across different breast cancer subtypes, such as invasive ductal carcinoma, invasive lobular carcinoma, and triple-negative breast cancer (104). Certain subtypes, particularly rare or aggressive ones, may be underrepresented in training datasets, leading to poor model performance. Errors in detecting these subtypes could result in missed diagnoses or misclassification, which is particularly concerning given the potential for delayed or inappropriate treatment. By categorizing errors by subtype, researchers can identify which cancer types are most challenging for the model and explore targeted approaches, such as incorporating more balanced datasets or developing subtype-specific models to improve detection rates.

In a multi-modal approach, different data types-such as histopathology images, mammograms, MRI scans, genomic sequences, and patient clinical histories-contribute unique information to the diagnostic process. However, each modality also presents its own set of challenges. For example, imaging data may suffer from noise, variability in acquisition protocols, or differences in resolution, affecting the model's ability to accurately detect tumors (105). Similarly, genomic data might be incomplete or noisy, leading to errors in models that rely heavily on this modality. Conducting an error analysis that dissects the performance by each modality allows for a clearer understanding of where the models excel and where they are prone to failure. This analysis can reveal if a model is overly reliant on one modality and potentially missing critical cues from others, suggesting a need for better data integration or improved feature extraction techniques.

Understanding the distribution and causes of false positives and false negatives is crucial for refining model performance (106). In breast cancer detection, false negatives-where the model fails to identify a cancerous lesion-pose significant risks, as they can lead to missed diagnoses and delayed treatment. False positives, on the other hand, may result in unnecessary biopsies, increased patient anxiety, and higher healthcare costs. Analyzing the circumstances under which these errors occur, such as specific imaging artifacts or ambiguous genomic markers, can provide insights into model weaknesses. For instance, if a high rate of false negatives is observed in certain mammogram images with dense breast tissue, this could indicate a need for more advanced image processing techniques or the inclusion of complementary data modalities to improve detection sensitivity.

Data quality and variability are significant factors influencing model performance (107). Inconsistent or poor-quality data, such as low-resolution images, incomplete clinical records, or non-standardized genomic data, can contribute to errors. Analyzing how variations in data quality affect model predictions can help identify the most impactful sources of noise or bias. This understanding can drive efforts to standardize data acquisition protocols, implement more rigorous data preprocessing steps, or develop robustness-enhancing strategies such as data augmentation or adversarial training.

The findings from a detailed error analysis can provide valuable insights for model improvement. For example, understanding which subtypes are most frequently misclassified or which modalities contribute to the majority of errors can guide the development of more focused and effective model architectures (108). Additionally, error analysis can inform the need for enhanced data fusion strategies that better leverage the strengths of each modality while mitigating their respective weaknesses. By continuously iterating on these insights, researchers can refine AI models to achieve higher accuracy, better generalizability, and more robust performance in clinical settings.

Integrating a comprehensive error analysis into the evaluation of multi-modal breast cancer detection models significantly enhances the understanding of model performance and robustness. By systematically examining errors by cancer subtype, modality, type of mistake, and data quality, researchers can identify critical areas for improvement and guide the development of more effective and reliable AI models (109). This approach not only strengthens the conclusions drawn from the study but also contributes to the advancement of AI-driven diagnostics, ultimately leading to better patient outcomes in breast cancer care.

#### 4.2.3 Novel trends in multi-modal techniques

Multimodal fusion is a crucial technique in multimodal analysis, involving the integration of multiple data sources to improve analytical capabilities. This approach is just one of several techniques in multimodal analysis, which explores the interactions between different types of data to achieve more comprehensive and nuanced insights. The integration of image and textual data has led to innovative applications in various fields, such as report generation, Visual Question Answering (VQA), cross-modal retrieval, and semantic segmentation.

The integration of multimodal data in medical informatics is a significant advancement, combining medical images and textual descriptions to generate comprehensive reports. This process streamlines clinical workflows and improves medical documentation accuracy. The process reduces clinician burden and ensures consistency and comprehensiveness in medical records (110). Visual Question Answering (VQA) is a field that uses multimodal integration to answer queries based on image data, particularly in medical contexts. It can interpret complex histopathology images and provide insights based on textual questions. Hartsock and Rasool (111) demonstrate the application of VQA in medical imaging, where a system trained on both image and text data can effectively answer questions about medical image content. This capability enhances diagnostic accuracy and facilitates educational tools in medical training. Cross-modal retrieval involves searching for information across different data modalities, such as histopathology, to retrieve relevant textual reports or case studies based on visual similarities in histopathology images (112).

Semantic segmentation is a technique that categorizes individual pixels in an image into meaningful categories, often using both image and text data. This technique can improve the segmentation accuracy in medical images by incorporating textual annotations for more precise and reliable results (113). Multimodal methodologies have gained significant scholarly attention in the medical field, particularly in leveraging medical images and textual data for improved diagnostic outcomes. Sun et al. (1) conducted a comprehensive scoping review of multimodal approaches in medical research, highlighting the growing interest in integrating various data types to enhance diagnostic accuracy and patient care. These methodologies have been instrumental in advancing personalized medicine, enabling more accurate diagnoses, and facilitating the development of tailored treatment plans.

## 5 An insight into explainable breast cancer diagnosis

Explainability is a crucial challenge in breast cancer detection, especially with the growing use of complex machine learning and deep learning models. It is essential for clinical decision-making, trust, transparency, regulatory compliance, and error detection. Explainable models help clinicians understand a diagnosis's rationale, fostering more informed decision-making and trust in automated systems. However, challenges include the complexity of models, data diversity, the black-box nature of algorithms, the trade-off between explainability and accuracy, and the lack of standardization in medical diagnostics. The absence of universally accepted standards leads to approach variability, complicating comparisons and consistent interpretations. AI systems used in breast cancer diagnosis often lack transparency, leading to inaccuracies in diagnosing breast cancer across different populations.

**Figure 5:**
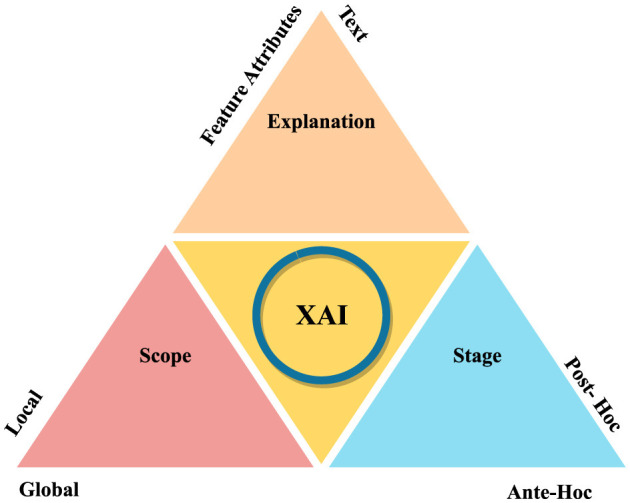
Different types of XAI methods.

Figure 5 illustrates how XAI methods can be categorized in different contexts. Based on Explanation, stage and scope, there can be different methods. Exaplaiability explanations can be in terms of feature attributes and textual format. In scope-based categorization, there are local and global methods. *Post-hoc* and *ante-hoc* are the stage-based XAI methods. Local and global methods offer specific insights into individual decisions, while intrinsic and *post-hoc* methods provide detailed explanations for black-box models. However, these methods may sacrifice complexity for interpretability, potentially reducing model performance. Model-specific and model-agnostic methods offer advantages and disadvantages, respectively.

Local methods provide specific insights into individual decisions, while global methods offer an overarching understanding of the model's behaviour across the dataset. However, they cannot generalize across different inputs and may overlook specific decision-making nuances. Model-specific methods can delve deep into a model's structure, while model-agnostic methods are flexible and can be used across different models without understanding their internal mechanics. However, model-specific methods are not transferable across different models and may offer less detailed explanations. Challenges such as data availability, diversity, semantic heterogeneity, and potential biases in explanations can affect the efficiency and acceptance of XAI methods.

**Figure 6:**
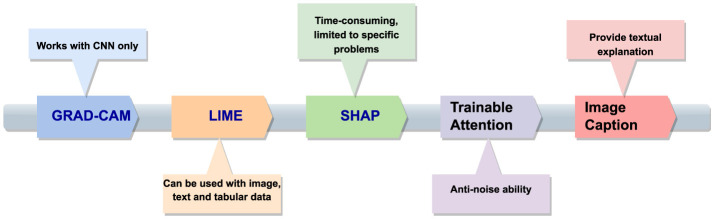
Recent XAI techniques.

XAI techniques can include Gradient-weighted Class Activation Mapping (GRAD-CAM), SHapley Additive exPlanations (SHAP), Local Interpretable Model-agnostic Explanations (LIME), Trainable Attention, and Image Caption. Figure 6 illustrates these methods with their features. XAI techniques, such as LIME and SHAP, offer local interpretations for understanding individual predictions and are model-agnostic, working across various models.

To enhance explainability, techniques such as feature importance and saliency maps can provide insights into which aspects of the input are driving the model's predictions. Model-agnostic methods like LIME and SHAP allow for the explanation of any machine learning model, offering flexibility in creating explainable outputs. Interpretable models, such as decision trees or linear models, provide greater transparency, albeit potentially at the cost of reduced accuracy. Additionally, human feedback and oversight in the diagnostic process can help validate and explain automated decisions, combining algorithmic efficiency with human intuition. In conclusion, explainability is crucial in breast cancer detection to ensure reliable and trustworthy outcomes, requiring a combination of technical solutions, regulatory compliance, and human oversight to create models that are both accurate and transparent.

LIME (114) is a technique employed to elucidate predictions made by complex machine learning models in the context of breast cancer detection. LIME provides local insights, making it easier to understand individual predictions and is particularly effective with smaller datasets such as gene clusters. Its model-agnostic nature allows it to be applied to various models, thereby offering versatility across different types of data. This flexibility helps clinicians and patients gain confidence in the diagnostic results produced by AI, enhancing trust in AI-driven diagnostic processes.

However, LIME is primarily limited to local explanations and may not provide a comprehensive understanding of the model's global behaviour. This limitation can be especially challenging in multi-modal data scenarios, where a holistic view of the model's decision-making process is crucial. Furthermore, the accuracy of LIME's explanations can be influenced by the perturbations generated around the instance being explained, potentially failing to capture the model's complexity accurately. Despite these limitations, LIME remains a valuable tool for enhancing the interpretability of AI in breast cancer detection, particularly in multi-modal contexts. By making the predictions of complex models more understandable, LIME significantly contributes to the transparency and trustworthiness of AI applications in medical diagnostics.

SHAP (115) is a sophisticated machine learning tool that assigns importance values to each feature for a specific prediction, thus elucidating how each feature contributes to the outcome. This tool is model-agnostic, meaning it can be applied to any machine learning model, offering considerable flexibility. SHAP provides both global and local explanations, delivering detailed insights into the overall model behaviour as well as individual predictions. It ensures consistency and reliability by accurately reflecting changes in the model's predictions through the SHAP values. Moreover, it adeptly handles missing features by setting their SHAP values to zero.

Despite its powerful capabilities, SHAP comes with certain drawbacks. It is computationally intensive, particularly for models with a large number of features, which can limit its feasibility in real-time applications. Additionally, interpreting SHAP values can be challenging for individuals without a technical background, which may hinder its broader accessibility. Nevertheless, SHAP remains a highly effective tool, especially for tree-based models like XGBoost, where understanding the impact of each feature is crucial. By enhancing the interpretability of AI models, SHAP contributes significantly to making these models more transparent and trustworthy for users. It provides a comprehensive framework for detailed analysis, facilitating a deeper understanding of how features influence outcomes and thereby improving the overall trust in machine learning predictions.

In the context of breast cancer diagnosis, SHAP's applicability extends to multi-modal data, integrating various data types such as histopathological images, genomic data, and clinical records. This integration enhances the model's robustness and provides a comprehensive understanding of the disease. By using SHAP to analyze multi-modal data, researchers can uncover intricate patterns and relationships that might be overlooked when considering a single data type. This holistic approach not only improves diagnostic accuracy but also aids in identifying key biomarkers and prognostic factors, ultimately contributing to more personalized and effective treatment strategies for breast cancer patients. Despite the computational challenges, the detailed insights provided by SHAP make it an invaluable tool in the complex landscape of multi-modal breast cancer diagnosis.

Class Activation Mapping (CAM) (116) is a fundamental tool in convolutional neural networks (CNNs) that generates heatmaps to visualize important parts of an image. Grad-CAM (117), an extension of CAM, uses gradients of any target concept to produce a coarse localization map, highlighting key regions for predicting concepts without requiring model modification or retraining. Grad-CAM is model-agnostic and applicable to various CNN models, making it valuable in tasks such as image classification and particularly useful in healthcare for identifying cancerous tissues in breast cancer diagnosis. However, Grad-CAM can sometimes produce too coarse localization, potentially prioritizing clinically irrelevant features and leading to false positives or incorrect interpretations. Grad-CAM++ improves upon Grad-CAM by providing finer localization and the ability to highlight multiple objects of interest in an image, making it better suited for explaining instances with multiple objects. Despite its advantages, Grad-CAM++ is more complex to implement and interpret. Integrating visual explanation methods like Grad-CAM and Grad-CAM++ into multi-modal data analysis presents challenges, particularly in ensuring coherent explanations across different types of data. Additionally, Grad-CAM-like methods are limited to CNN models, restricting their use in non-CNN models and potentially complicating decision-making processes in those contexts.

Trainable attention in Explainable Artificial Intelligence (XAI) is an advanced technique that emphasizes critical features in input data, such as images or text, to make predictions (118). This approach is particularly valuable in multi-modal breast cancer diagnosis, where models must analyze diverse data sources including mammograms, ultrasounds, and patient histories. By focusing on the most relevant features, trainable attention enhances interpretability for clinicians, improves diagnostic accuracy, and can be customized to specific types of data. In the context of multi-modal breast cancer diagnosis, trainable attention can integrate and prioritize information from various modalities, leading to a more comprehensive understanding of the patient's condition. For instance, it can highlight specific regions in a histopathology image that correlate with textual descriptions from patient histories, thereby providing a clearer picture of potential issues.

Despite its advantages, trainable attention faces several challenges. One major issue is the complexity of implementing such models, which require sophisticated algorithms and significant computational resources. Additionally, there is a risk of overemphasizing certain features, potentially leading to biased predictions. Implementation challenges also include ensuring the system's robustness and generalizability across different patient populations and clinical settings. Nevertheless, trainable attention (119) remains a promising approach to enhancing model interpretability and focus in multi-modal breast cancer diagnosis. By addressing the challenges associated with its implementation, the full potential of trainable attention can be realized, thereby advancing the accuracy and reliability of AI-driven diagnostic tools. In conclusion, while trainable attention in XAI offers significant benefits in improving the interpretability and accuracy of multi-modal breast cancer diagnosis, ongoing efforts to overcome its inherent challenges are essential. Through continuous development and refinement, this technique holds the potential to become an integral component of advanced diagnostic systems, ultimately contributing to better patient outcomes.

Image captioning in XAI involves generating descriptive text for images, which aids in elucidating the decision-making process of AI models (120). This technique is particularly relevant in the context of multi-modal breast cancer diagnosis, where it can provide significant benefits. Image captioning offers an easy-to-understand explanation of what the model detects in medical images, such as mammograms or ultrasounds, thereby making the AI's decision-making process more transparent. By translating complex patterns into textual descriptions, image captioning facilitates better communication between AI systems and medical professionals, enhancing the collaborative diagnostic process. This method also makes the findings of AI models more accessible to non-specialists, including patients, by providing explanations in natural language, which helps in understanding the diagnosis and treatment options.

However, the utility of the generated captions is heavily reliant on the accuracy of the underlying model. Errors in interpretation by the model can result in misleading captions, potentially affecting diagnostic decisions. Incorporating image captioning into multi-modal diagnostic systems presents challenges, as it requires the model to accurately understand and explain data from various sources, ensuring coherent and accurate descriptions. Furthermore, the simplification necessary for generating captions might omit critical details, leading to oversimplified explanations that could overlook nuances essential for an accurate diagnosis. In summary, while image caption XAI methods hold promise for enhancing the interpretability and accessibility of AI in breast cancer diagnosis, their implementation must be meticulously managed to prevent misinterpretation and oversimplification. Proper integration and careful validation are essential to fully leverage their potential in clinical settings.

### 5.1 Existing methods: an explainability perspective

In the domain of uni-modal breast cancer detection, significant advancements have been made in integrating explainability techniques to enhance the interpretability and reliability of predictive models. Gu et al. (121) developed an auxiliary decision support system that combines ensemble learning with case-based reasoning (CBR) to predict breast cancer recurrence. Using XGBoost for predictions and CBR to provide comprehensible explanations, this system effectively communicated the importance of various attributes, aligned well with human reasoning, and gained acceptance among clinicians. Kabakçı et al. (122) proposed an automated method for determining CerbB2/HER2 scores from breast tissue images by adhering to ASCO/CAP recommendations. This method employed cell-based image analysis and a hand-crafted feature extraction approach, ensuring both interpretability and adaptability to guideline updates without the need for re-training.

Moreover, recent studies have focused on enhancing the explainability of deep learning models used in breast cancer histopathology. Maleki et al. (62) utilized pre-trained models combined with gradient-boosting classifiers to achieve high accuracy in classifying breast cancer images from the BreakHis dataset. Similarly, Peta and Koppu (123) introduced an explainable deep learning technique involving adaptive unsharp mask filtering and the Explainable Soft Attentive EfficientNet (ESAE-Net), which provided improved visualization and understanding of classification decisions. Jaume et al. (124) presented CGEXPLAINER, a *post-hoc* explainer for graph representations in digital pathology, which pruned redundant graph components to maximize mutual information between the original prediction and the sub-graph explanation. These contributions, along with methods like the cost-sensitive CatBoost classifier with LIME explainer (125) and the use of SHAP for feature importance analysis in tumor cellularity assessment (126), highlight the growing emphasis on explainability to ensure that AI systems for breast cancer detection are not only accurate but also interpretable and trustworthy for clinical application.

Explainability is a critical factor in radio genomics (127), as it fosters trust with end-users like physicians and patients, driving the deployment of deep learning models in research and clinical practice. It increases confidence in the model's decision-making process, enabling better understanding and acceptance of results. Explainability also serves as a debugging process for model training and fine-tuning, identifying potential errors or biases. It also helps bypass malicious manipulation, ensuring the integrity and security of radiogenomic research and its applications. In the healthcare field, explainability is especially important as it facilitates better interpretation and understanding of complex AI models, leading to improved patient care and treatment outcomes.

Holzinger et al. (128) proposed the utilization of Graph Neural Networks (GNNs) as a method for achieving multi-modal causability within XAI (xAI). This approach facilitated information fusion through the establishment of causal links between features using graph structures. The method's objective was to construct a multi-modal feature representation space, utilizing knowledge bases as initial connectors for the development of novel explanation interface techniques. Essential components included intra-modal feature extraction and multi-modal embedding. Various GNN architectures and graph embeddings, such as GCNN, Graph Isomorphism Network (GIN), and SchNet, were considered viable options. Additionally, dynamic GNN architectures like Pointer Graph Networks (PGN) were employed to enable the processing of adaptive graphs. Zhang et al. (129) introduced a Deep Multimodal Reasoning and Fusion Network (DMRFNet) for Visual Question Answering (VQA) and explanation generation. The model employed multimodal reasoning and fusion techniques to improve the accuracy of answers and explanations. A key innovation was the Multi-Graph Reasoning and Fusion (MGRF) layer, which utilized pre-trained semantic relation embeddings to handle complex spatial and semantic relations among visual objects. DMRFNet was capable of being stacked in depth to facilitate comprehensive reasoning and fusion of multimodal relations. Additionally, an explanation generation module was incorporated to provide justifications for predicted answers. Experimental findings demonstrated the model's effectiveness in achieving both quantitative and qualitative performance improvements.

Kang et al. (130) introduced a segmentation framework with an interpretation module that highlights critical features from each modality, guided by a novel interpretation loss with strengthened and perturbed fusion schemes. This approach effectively generates meaningful interpretable masks, improving multi-modality information integration and segmentation performance. Visualization and perturbation experiments validate the effectiveness of the interpretation method in exploiting meaningful features from each modality. An interpretable decision-support model for breast cancer diagnosis using histopathology images was proposed in Krishna et al. (131). This method integrated an attention branch into a variant of the DarkNet19 CNN model to enhance interpretability and performance. The attention branch generated a heatmap to identify regions of interest, while the perception branch performed image classification through a fully connected layer. Training and validation utilized over 7,000 breast cancer biopsy slide images from the BreaKHis dataset, resulting in a binary classification accuracy of 98.7%. Notably, the model offered enhanced clinical interpretability, with highlighted cancer regions corresponding well with expert pathologist findings. The ABN-DCN model effectively combined an attention mechanism with a CNN feature extractor, thereby improving both diagnostic interpretability and classification performance in histopathology images.

Evaluation of XAI (Explainable AI) techniques such as Grad-CAM, SHAP, and LIME in clinical settings requires a comprehensive assessment framework, focusing on both their technical performance and practical application. There are several key metrics that should be defined, including fidelity, which measures how accurately explanations reflect the model's decision-making process, and interpretability, which is measured by how easy it is for clinicians to understand these explanations, often through Likert scale ratings. Metrics like localization accuracy are used to evaluate how well highlighted regions, as identified by Grad-CAM, correspond with relevant clinical areas. In addition, feature importance consistency, particularly for SHAP and LIME, is also essential for ensuring stable and reliable explanations across different cases, thereby fostering trust in the model. In order to provide a broader perspective on transparency, an explainability score combines aspects such as model simplicity and clarity, in order to evaluate how well AI model predictions align with clinical practice. Additionally, human-AI agreement and time efficiency metrics are used to assess alignment with clinical judgment and the ease of interpreting explanations. Through user studies and surveys with clinicians, as well as scenario-based testing, comprehensive feedback is obtained. By comparing the XAI methods with standard clinical practices and assessments across different settings, this structured evaluation ensures robustness, transparency, and reliability, thus enhancing the clinical utility of the methods.

### 5.2 Latest XAI approaches in multi-modal context

X-VARS (132), a multimodal large language model initially designed for football refereeing tasks, utilized Video-ChatGPT to process video features and predict responses. This model emphasized interpretability and has demonstrated strong performance in human studies, indicating its potential for adaptation in breast cancer detection. By integrating diverse data sources, such as histopathology images and clinical records, similar models could offer comprehensible diagnostic support, thereby enhancing the accuracy and transparency of the diagnostic process. The LeGrad (133) explainability method, which employs Vision Transformers (ViTs) (134), utilizes techniques such as GradCAM (117) and AttentionCAM (135) to provide granular insights into feature formation. These explainability methods are crucial for breast cancer detection, offering transparent interpretations of model decisions. By adapting this method to a multimodal scenario that includes histopathology images, and clinical or textual data, can provide comprehensive diagnostic support. This integration enhances trust and clinical applicability by offering transparent and interpretable insights across various data types, thereby improving the accuracy and reliability of breast cancer diagnostics.

The method proposed by Hu et al. (112) for fine-grained cross-modal alignment between histopathology WSIs and diagnostic reports holds promise as a future avenue in explainable multimodal breast cancer detection. By leveraging anchor-based WSI and prompt-based text encoders, this method ensured that relevant diagnostic information was accessible and interpretable to pathologists. Through precise alignment and interpretation of multimodal diagnostic data, including histopathology images and clinical and textual reports, the method enhances transparency and interpretability in breast cancer diagnosis. This approach can provide clear insights into the decision-making process of diagnostic models, thereby enhancing trust and clinical acceptance in the application of multimodal AI systems for breast cancer detection. A multimodal image search strategy was described in Tizhoosh and Pantanowitz (136) as a method of improving diagnosis, prognosis, and prediction in histopathology. With this method, large image archives can be explored to identify patterns and correlations using foundation models for feature extraction and image matching. A breast cancer detection framework based on this framework could provide efficient retrieval and comparison of histopathology images, thereby aiding in the identification of malignancies and their characteristics.

Investigating local surrogate explainability techniques in deep learning models, researchers explored the use of VisualBERT and UNITER networks to generate multimodal visual and language explanations (137). The potential of these models to mimic domain expertise underscores the value of XAI techniques in breast cancer detection. By providing clear and understandable rationales for automated decisions, such methods enhance clinical trust and support informed decision-making in diagnostic processes. A framework named LangXAI (138) was introduced, integrating XAI with advanced vision models to generate textual explanations for visual recognition tasks. This framework enhances transparency and plausibility, potentially improving breast cancer detection by making the diagnostic process more understandable and reliable for clinicians. Consequently, it supports better patient outcomes.

Various XAI methods, including Gradient backpropagation and Integrated-Gradients, were applied in Rehman Hashmi et al. (139) to analyze the MedCLIP model. These methods provided valuable insights into model predictions, offering pivotal information for the development of breast cancer detection models. Ensuring the transparency and comprehensibility of model decisions can play a crucial role in facilitating regulatory compliance and fostering clinical acceptance of such models in diagnostic settings. A tool called LVLM-Interpret (140) was developed to interpret responses from large vision-language models, employing techniques such as raw attention and relevancy maps. This tool's capacity to visualize and comprehend model outputs can be utilized in breast cancer detection to improve the interpretability and reliability of AI-driven diagnostic tools.

An ex-ILP framework was introduced to enhance reasoning capabilities in vision-language models by Yang et al. (141). By improving implicit reasoning skills, this methodology could be harnessed in breast cancer detection to interpret complex interactions between visual and textual data, thus contributing to more accurate and nuanced diagnostic insights. The NLX-GPT method, introduced in Sammani and Deligiannis (142), integrated discriminative answer prediction and explanation tasks into a unified model. This approach, which achieves high performance across diverse tasks, holds the potential for adaptation in breast cancer detection. By providing both diagnostic conclusions and their explanations, the NLX-GPT method enhances the usability and trustworthiness of AI models in clinical settings.

## 6 Exploring future directions in multi-modal explainable for breast cancer diagnosis

Multi-modal data integration in histopathology enhances diagnostic accuracy and robustness by combining diverse data modalities from the same patient. In breast cancer detection, these modalities include histopathology images, radiological scans, genomic data, and textual clinical reports. Acquiring comprehensive multi-modal datasets presents challenges (143) due to the varied nature of data, high cost and complexity of data collection, and difficulties in synchronizing and correlating data across different modalities. The scarcity of comprehensive multi-modal datasets has led to the exploration of synthetic data generation techniques, such as Generative Adversarial Networks (GANs) (144) and Variational Autoencoders (VAEs), which create realistic data to augment existing datasets and provide diversity for training robust machine learning models (145).

Text-to-image synthesis, where descriptive text is converted into corresponding images, is an emerging field with significant implications for histopathology. This approach can generate detailed histopathology images from textual descriptions of patient pathology reports. For instance, Reed et al. (146) demonstrated the capability of GANs to generate high-resolution images from textual descriptions, which can potentially be adapted to create synthetic histopathology slides from clinical narratives. This methodology not only aids in dataset augmentation but also in visualizing pathological conditions described in text format, thereby bridging the gap between clinical reports and image data. Conversely, image-to-text generation involves converting visual data, such as histopathology images (110), into descriptive text. This technique can automate the generation of pathology reports from histological images, thereby reducing the workload of pathologists and improving the consistency of diagnostic reports.

Modality conversion in multi-modal histopathology is a crucial area of research, enabling the integration of complementary information from different imaging modalities. GAN-based models have shown promise in this domain, allowing the transformation of medical images across different modalities while preserving anatomical structures (147, 148). This is particularly relevant for creating histopathology images from non-invasive imaging techniques, reducing the need for invasive biopsies (147). Cross-modal data generation techniques, such as CycleGAN, can synthesize one type of imagery from another, generating histopathology-like images from non-histopathology data sources. However, challenges (149) remain, such as ensuring the fidelity and clinical relevance of synthetic data, as inaccuracies can lead to erroneous model training and diagnostic conclusions. Additionally, robust validation is needed to ensure the integration of generated data into existing workflows meets necessary clinical standards and regulations.

Future research should focus on refining generative models to enhance the quality and realism of synthetic data, especially in histopathology. Techniques that integrate synthetic data with real-world clinical data are crucial for advancing multi-modal breast cancer detection. Exploring novel generative methods, such as combining genomic and imaging data, can enhance the richness and utility of multi-modal datasets, leading to more accurate and comprehensive diagnostic tools. This approach can address data scarcity challenges and improve the robustness of multi-modal breast cancer detection systems. This will lead to improved accuracy, transparency, and patient outcomes in histopathology-based breast cancer diagnosis.

### 6.1 Advancing toward a new framework

**Figure 7:**
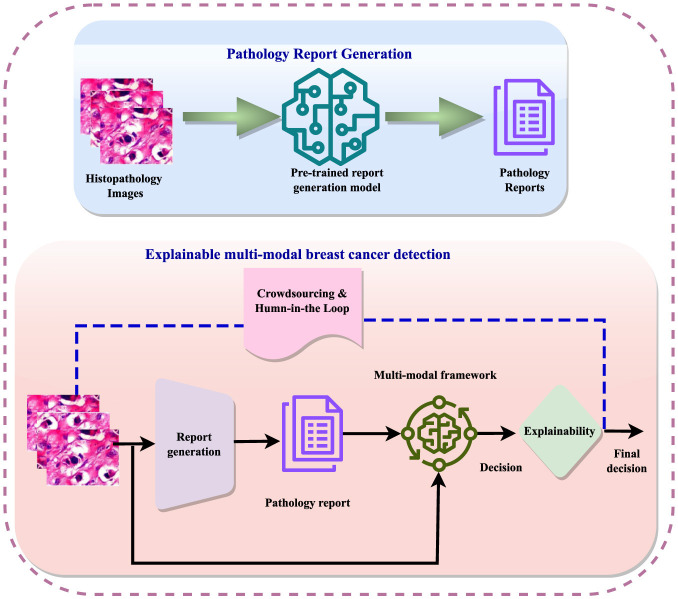
Proposed framework for explainable multi-modal breast cancer detection.

The framework proposed for multimodal explainable breast cancer diagnosis involves a systematic process aimed at enhancing diagnostic accuracy and transparency while integrating human expertise for improved patient outcomes. Figure 7 illustrates the proposed framework. In the initial step, histopathology images are processed using pre-trained medical report generation models. These models, such as CLARA (150), automatically generate comprehensive reports from the images, augmenting them with relevant features extracted through computer vision techniques. Clinical data, including patient history and laboratory results, are integrated into the report generation pipeline to ensure contextually relevant diagnostic reports.

Subsequently, in the multimodal explainable framework for diagnosis, the generated diagnostic reports and histopathology images serve as input. Visual language models, such as Vision Transformers or large language models, are employed to process both visual and textual information simultaneously. Explainability techniques like GradCAM and AttentionCAM are implemented to provide interpretable insights into model decisions, enhancing transparency and trust in the diagnostic process. Model outputs are visualized using tools like LVLM-Interpret to improve interpretability and reliability.

Human expertise is integrated through crowdsourcing or expert consultations to validate and refine model predictions, ensuring clinical relevance and accuracy. This human-in-the-loop approach facilitates informed decision-making and iterative refinement based on feedback from clinicians and patients. Ultimately, the framework supports diagnostic support by providing transparent and understandable diagnostic conclusions, along with explanations for model predictions. It is integrated into existing clinical workflows to streamline diagnostic processes and enhance patient care, contributing to advancements in the field of breast cancer diagnostics.

## 7 Conclusion

Breast cancer diagnosis has evolved significantly with the advent of multi-modal methodologies, which combine histopathology images with non-image data. These methods offer a more comprehensive view of breast cancer pathology, enhancing diagnostic confidence and accuracy. The use of Explainable Artificial Intelligence (XAI) in multi-modal diagnoses highlights the importance of transparency in diagnostic procedures. Despite challenges like computational complexity and the need for coherent explanations across different data types, the potential of XAI in multi-modal contexts is significant. The review also advocates for the development of new frameworks that leverage advanced AI techniques while ensuring interpretability. These advancements aim to address existing limitations and develop personalized treatment strategies tailored to each patient's unique needs. By leveraging multi-modal data and emphasizing explainability, these methods enhance diagnostic accuracy, bolster clinician confidence, and foster patient engagement. In conclusion, the integration of multi-modal data and explainable AI techniques represent significant advancements in breast cancer diagnosis. By overcoming the constraints of uni-modal approaches and enhancing the interpretability of diagnostic models, these methods hold promise for improving diagnostic accuracy, patient outcomes, and clinician trust in AI-driven healthcare solutions. This review contributes to a comprehensive understanding of multi-modal diagnostic techniques and the imperative of explainability, informing strategic directions in breast cancer diagnosis and treatment, ultimately striving for improved patient outcomes and a more effective healthcare landscape.
